# Modeling Cell-to-Cell Communication Networks Using Response-Time Distributions

**DOI:** 10.1016/j.cels.2018.01.016

**Published:** 2018-03-07

**Authors:** Kevin Thurley, Lani F. Wu, Steven J. Altschuler

**Affiliations:** 1 Department of Pharmaceutical Chemistry, University of California San Francisco, San Francisco, CA 94158, USA

## Abstract

Cell-to-cell communication networks have critical roles in coordinating diverse organismal processes, such as tissue development or immune cell response. However, compared with intracellular signal transduction networks, the function and engineering principles of cell-to-cell communication networks are far less understood. Major complications include: cells are themselves regulated by complex intracellular signaling networks; individual cells are heterogeneous; and output of any one cell can recursively become an additional input signal to other cells. Here, we make use of a framework that treats intracellular signal transduction networks as “black boxes” with characterized input-to-output response relationships. We study simple cell-to-cell communication circuit motifs and find conditions that generate bimodal responses in time, as well as mechanisms for independently controlling synchronization and delay of cell-population responses. We apply our modeling approach to explain otherwise puzzling data on cytokine secretion onset times in T cells. Our approach can be used to predict communication network structure using experimentally accessible input-to-output measurements and without detailed knowledge of intermediate steps.

## In Brief

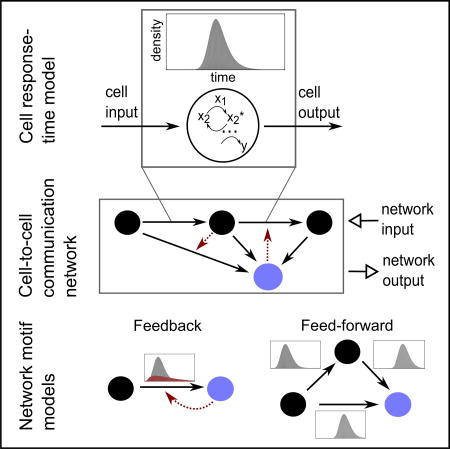

Interacting cellular communities have critical roles in biological functions such as tissue development or immune responses. Cell-to-cell communication networks comprise both intra- and intercellular processes, making detailed mathematical models intractable. Here, we develop a scalable framework for modeling extra-cellular communication networks that treats intracellular signal transduction networks as “black boxes” with characterized input-to-output response relationships. We discover that a range of dynamic cell-population behaviors, including cellular synchronization, delays, and bimodal responses, can emerge from simple cell-to-cell communication networks.

## INTRODUCTION

In multicellular organisms, cells live in communities and constantly exchange signaling molecules. Prominent examples of short-range communication are diffusible ligands shaping immune responses (Schwartz et al., 2015) and the tumor microenvironment (Balkwill et al., 2012), notch-delta-mediated signals (Guruharsha et al., 2012), and microvesicles (Raposo and Stoorvogel, 2013). In the mammalian immune system, cell-to-cell communication can involve multiple cell types (e.g., T cells, neutrophils, macrophages, and epithelial cells) communicating through tens of different types of cytokine species (Burmester et al., 2014; Schwartz et al., 2015). In many cases, cytokines secreted by one cell type act in a relay on other cell types, as well as affect the original cell type. An important example is interferon gamma (IFN-γ), which is secreted by Th1 cells (a subclass of T cells), stimulates macrophages, and also induces the differentiation of T cells toward Th1 cells. The levels of various cytokine species vary by an order of magnitude or more between supernatants of isolated cells and cell populations (Schrier et al., 2016; Shalek et al., 2014; Xue et al., 2015), suggesting pronounced effects of cell-to-cell communication on the cytokine milieu.

Within a cell, extensive research has identified many molecules and pathways involved in signal transduction and, in many cases, has also developed an understanding of their function. In particular, the identification and analysis of generic network motifs has led to an understanding of how certain interaction topologies can function to suppress noise, amplify signals, or provide robustness (Alon, 2007; Alon et al., 1999; Heinrich et al., 2002; Hornung and Barkai, 2008; Shen-Orr et al., 2002). For this purpose, mathematical models of simplified systems have often been an important driving force, which have helped to reveal engineering principles such as feedback control and perfect adaptation (Altschuler et al., 2008; Fritsche-Guenther et al., 2011; Ma et al., 2009).

At the level of communication among cells, the mapping from general network motif to function is poorly understood. In cell-to-cell communication networks, each node is a type of cell and each type of cell processes input signals through intracellular networks to elicit an output; outputs are a cell-state change and (potentially) an input signal to other cell types or even its own cell type. Thus, cell-to-cell communication networks are complex: they are “networks of networks”; they can contain different cell types with different input-to-output relationships; the response times of cells—even within one type—to identical input stimuli is heterogeneous; and output of any one cell can recursively become an additional input signal to other cells.

Whereas the well-studied rules of chemical kinetics can be applied to model the building blocks of intracellular networks (e.g., proteins, metabolites, etc.), it is unclear how best to model cell-to-cell communication networks. Existing studies of cell-to-cell communication have largely focused on specific cases—such as the cytokines interleukin-2 (IL-2) (Feinerman et al., 2010; Fuhrmann et al., 2016; Thurley et al., 2015; Waysbort et al., 2013), IFN-γ (Helmstetter et al., 2015; Schulz et al., 2009), or tumor necrosis factor alpha (TNF-α) (Paszek et al., 2010; Tay et al., 2010). However, in most settings, most if not all intracellular network parameters are unmeasured or inaccessible with current experimental approaches. Thus, there is a need to develop more general approaches for investigating the behaviors of cell-to-cell communication networks.

Here we propose response-time modeling as a framework to unify and interpret knowledge on intra- and intercellular signaling pathways. In this framework, temporal input-to-output relationships of intracellular signaling networks are captured by “response-time distributions.” These distributions, which measure the probability over time of observing a cellular output to a given input, can be measured from experiment or estimated from theoretical models. Focusing on response-time distributions allows us to elide detailed descriptions of intermediate intracellular signaling steps and focus on emergent properties that arise at the population level from cell-to-cell communication. Below, we first characterize response-time distributions that can arise from intracellular networks and find that, in many cases, they can be well modeled by gamma distributions. Second, we use this observation to analyze common cell-to-cell communication network motifs, and discover that different interaction topologies can regulate a rich set of dynamic behaviors, including delayed, synchronized, and bimodal cellular responses to a stimulus. Finally, we apply our approach to investigate recent data on cytokine secretion onset times.

## RESULTS

### Response-Time Modeling of Cell-State Dynamics

**Figure 1:**
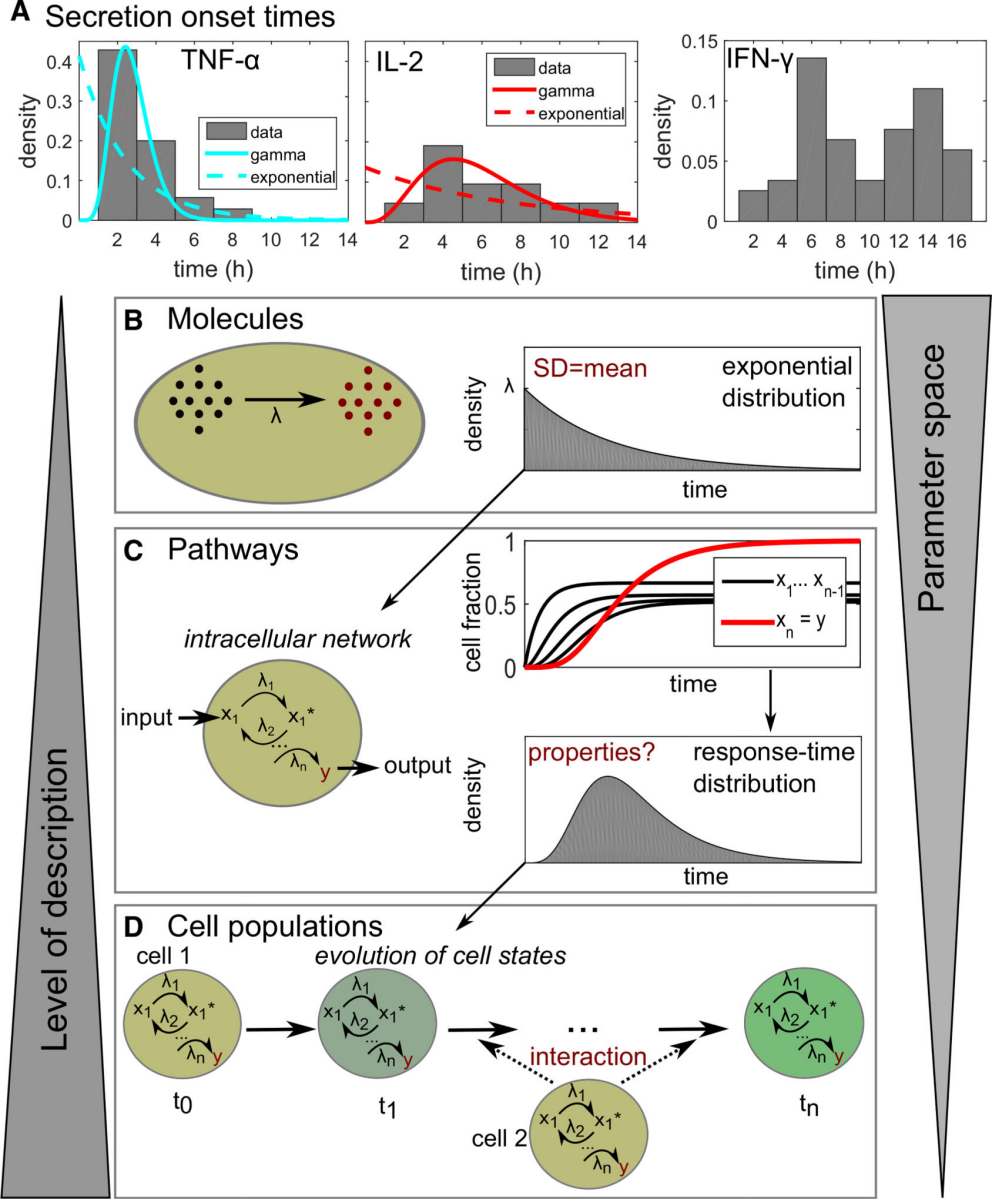
Response-Time Modeling of Cell-State Dynamics (A) Cytokine secretion onset times in CD8+ T cells from Han et al. (2012). Data were taken from the original publication and re-normalized to probability distributions. For TNF-α and IL-2, also best-fit curves to the gamma distribution and the exponential distribution are shown (fitting parameters in Table S1). (B) An elementary chemical reaction is well described by a simple rate equation, with a single rate parameter *λ* (concentration per time). However, the waiting time until the next reaction occurs is a random variable. Chemical reaction kinetics dictate that the response times are exponentially distributed. (C) Cellular state changes require a set of chemical reactions forming an intracellular reaction network. That network can be described by differential equations for each reaction, whose solutions reveals the fraction of cells containing each molecular species at every time point. From that information, we can calculate the response-time distribution for a cell state of interest. That response-time distribution does not need to be exponential or monotonic, but can have one or even several peaks. (D) The response of a cell population to a stimulus is often not only dependent on intracellular networks, but may also evolve by intercellular communication. Response-time modeling uses the response-time distributions for all considered cell-state changes, and their dependence on other cell states, to characterize the intercellular communication network.

Temporal heterogeneity has been widely observed for cellular processes that involve cell-to-cell communication, such as onset of cytokine secretion or cell differentiation (Figure 1A; Table 1). The timing of cellular responses often cannot be described by a simple Poisson process, which would characteristically show exponentially distributed response times (Figure 1B) (Gillespie, 1992). Rather, single-peaked and even bimodal distributions have been observed, reflecting the complex networks underling many biological processes.

We wondered whether cell-to-cell communication networks could be modeled and analyzed in a way that abstracts molecular detail yet still captures essential dynamic properties. By analogy, a full description of elementary chemical reactions requires knowledge of the positions and velocities of all molecules at all times (Gillespie, 1992); however, these reactions can be well approximated by a single phenomenological parameter, namely the reaction rate constant. For modeling cellular state changes, one must take into account that the response of a cell to an input signal is not a single-step reaction but rather a result of a multi-step intracellular reaction network (Figure 1C). Further, the response of the cell may also depend on input it receives from other cells that have processed their own input signals, changed state, and, consequently, produced their own signals. Experimentally, “cell state” is typically observed phenomenologically as distinct phenotypic states of a cell (e.g., based on threshold intensities of fluorescence markers).

The time elapsed until an observable cellular state-change happens—the response time—is a random variable, and its distribution across a cell population can be interpreted as a normalized probability distribution function (Box 1). In contrast to the times until the next molecular event in a single-step reaction (Figures 1B and 1C), the response times of cells are, in general, not exponentially distributed. Rather, the cellular response-time distribution is a signature function depending on and describing the relevant intracellular processes, with no known *a priori* properties (Box 1). The advantage of response-time modeling is that it focuses on a small number of key measurable events (Chevalier and El-Samad, 2009; Mastny et al., 2007), and the behaviors of cell populations can be described with a rather small number of parameters (Figure 1D).

### Simple Intracellular Networks Induce Single-Peaked Response-Time Distributions

In a literature survey, we found that many reports of experimentally measured response-time distributions indicate a single-peaked type of distribution. Such distributions have been reported for a wide range of cellular systems from gene transcription over cellular Ca^2+^ spikes to cytokine secretion (Table 1; Figure 1A). Notable exceptions are some processes where exponential distributions have been measured, and bimodal IFN-γ secretion onset times in T cells, which we discuss in detail later.

**Figure 2:**
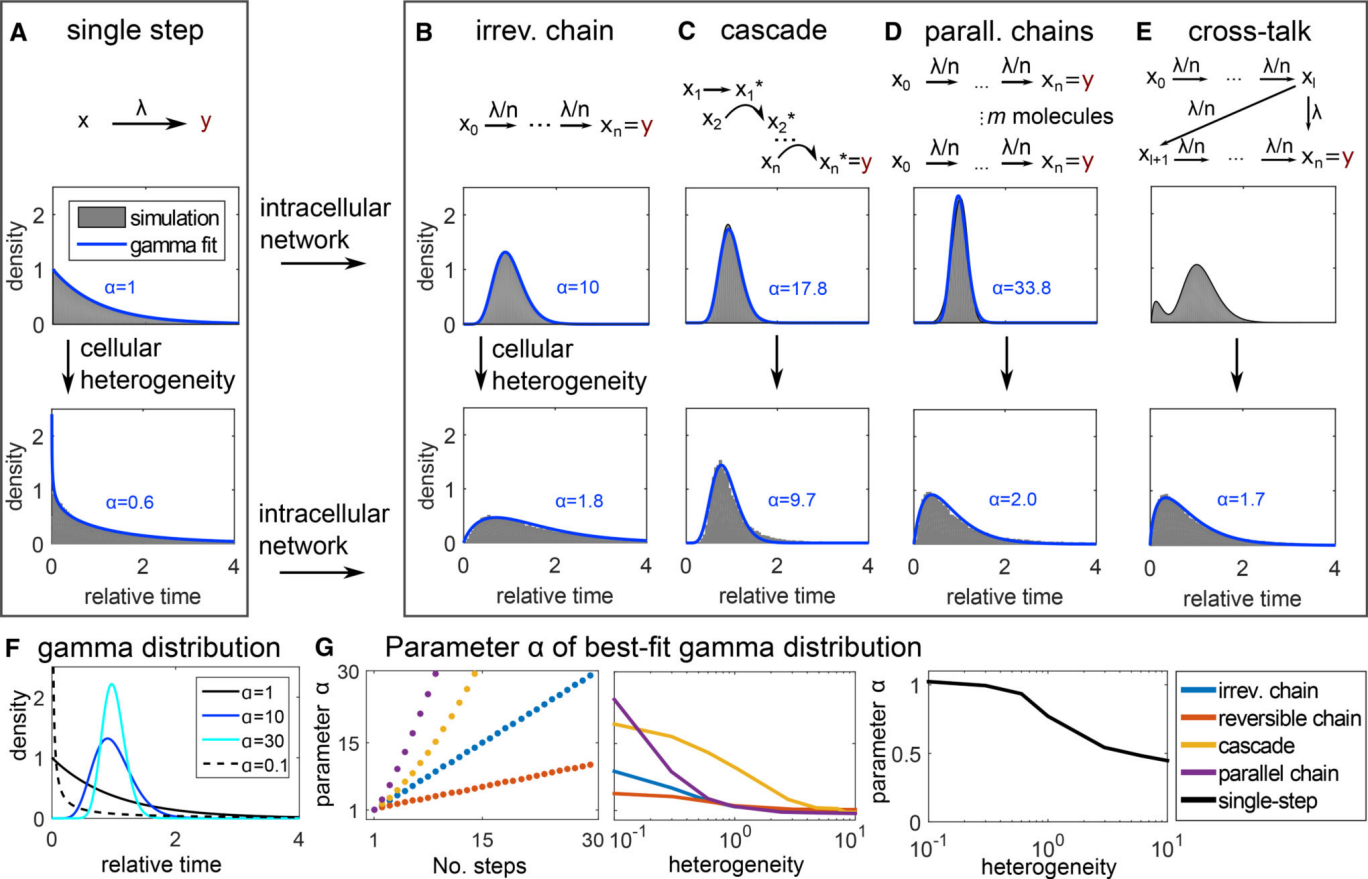
Intracellular Reaction Networks Are Often Well Described by Gamma-Distributed Response Times (A–E) Response-time distributions of the multi-step models shown in the top row of each panel. Top: each arrow represents an elementary (i.e., single-step) reaction (see STAR Methods for model equations). Response-time distributions are computed by solving the corresponding system of differential equations and normalizing by the distribution average. Bottom: to account for cellular heterogeneity, the rate parameter *λ* is drawn from a log-normal distribution (SD = mean), and normalized response-time distributions are obtained by stochastic simulation. For all models, heterogeneous *λ* results in longer tails and earlier peaks. Blue lines: best-fit gamma distributions, labels indicate the shape parameter *α*. Parameters: *n* = 10, *λ* = 1, *l* = 1. (F) Plots of the gamma distribution (Equation 1) with rate parameter *β* = 1/*α* (i.e., the average time is constant) and shape parameter as indicated. (G) Shape parameter *α* of best-fit gamma distributions to the indicated models (A–D) and (Figure S1A). “No. steps”: parameter *n* in the models. Cellular heterogeneity: coefficient of variation of the log-normal distribution generating *λ*.

Why does this widespread occurrence of single-peaked response-time distributions occur, and what does it mean for the typical dynamics of a cell population? Response times for single-step reactions are exponentially distributed (Figures 1B and 2A, top). However, cellular signal transduction typically is driven by intracellular networks comprising phosphorylation cascades, feedback, crosstalk, etc. As a simple illustration, consider a uniform, irreversible reaction chain, i.e., the cellular response is triggered after completion of *n* reaction steps all driven by the same rate constant *μ* = λ/*n* (Figure 2B, top). This process has the same average response time as a single reaction with rate λ, but the distribution *ψ_n_*(*t*) (see Box 1 for exact definitions) of the response times over a cell population changes: The process can be regarded as a sum of *n* single-step processes (elementary reactions), and therefore the over-all response time is the *n*-fold convolution (Van Kampen, 2002)

(Equation 1) $$\psi_{n}(t)=\underset{\text{n times}}{\underset{︸}{\left[\mu e^{-\mu t}*\mu e^{-\mu t}*\ldots \right]}}=\frac{t^{n-1}e^{-\mu t}\mu^{n}}{(n-1)!}=\gamma (n,\mu ;t).$$

Here, * denotes convolution and *γ*(*α, β; t*) is known as the gamma distribution with shape parameter *α* and rate parameter *β* (in general, *α* can take non-integer values, see the STAR Methods).

Indeed, the single-peaked response-time distributions observed experimentally can be described by gamma distributions (Table 1), as, for *α* > 1, the gamma distribution is an asymmetric (right-skewed) distribution with a single peak at *t* > 0. The observed exponential distributions for single-enzyme kinetics, offset of transcription, and intracellular Ca^2+^ puffs, indicate single-step processes (Figure 2A): All these processes are likely dominated by a single molecular reaction (binding of a metabolite to an enzyme, unbinding of a transcription factor from DNA, opening of a Ca^2+^ channel subunit).

Intracellular signaling pathways are usually not simple irreversible chains, and, therefore, we asked whether the observed single-peaked distributions can be generated by a broader class of intracellular network models. Indeed, single-peaked distributions have previously been reported for more realistic models of cellular signal transduction like kinetic proofreading (Bel et al., 2010), multiple phosphorylation (Lu et al., 2006), and Ca^2+^ signaling (Thurley et al., 2014). Here, we studied three additional simple network motifs in more detail: The signaling cascade (Heinrich et al., 2002) (Figure 2C), a set of parallel irreversible chains reflecting *m* receptor molecules that each can trigger a cellular response as a “race to the nucleus” (Lu et al., 2006) (Figure 2D), and the reversible chain (Figure S1A). The response times of all those examples are well approximated by gamma distributions (Figures 2C, 2D, and S1A, top, and S1B and S1C), although some fitting errors arise in models that tend to induce long tails (e.g., cascade model with high degree of heterogeneity, see Figure S1B).

Apart from intracellular networks, another complication is cellular heterogeneity: in a cell population, even a clonal one, we cannot expect that each cell has the same reaction rate for a certain intracellular process. Rather, gene expression and receptor expression levels show heterogeneity (Altschuler and Wu, 2010). To investigate the effect of such heterogeneity on the response-time distribution, we used log-normal distributed reaction-rate parameters (Figures 2A–2E and S1A, bottom). In all models, the response-time distribution shifts toward longer tails and earlier peaks after incorporating cellular heterogeneity, but is still well approximated by a gamma distribution (Figures 2A–2D and S1A, bottom, and S1B). This is expected: intuitively, adding cellular heterogeneity should reduce predictability—indeed, adding heterogeneity can never induce a peak in the single-step process and rather leads to long tails (STAR Methods). However, adding high numbers of intermediate intracellular steps increases the predictability of the process, due to the central limit theorem (Van Kampen, 2002); it tightens the peak in the response-time distribution (Figures 2F and 2G).

Thus far, we only studied unbranched multi-step processes. Finally, we considered crosstalk within an intracellular multi-step process (Figure 2E). In this case, a bimodal response-time distribution can occur, but, even here, heterogeneity of rate parameters shifts the distribution toward a gamma distribution (Figure 2E, bottom panel), offering another demonstration of the versatility of gamma distributions. Therefore, in the following discussion, we will focus on cell population responses that induce gamma-distributed response times.

### Response-Time Modeling of Intercellular Network Motifs

**Figure 3:**
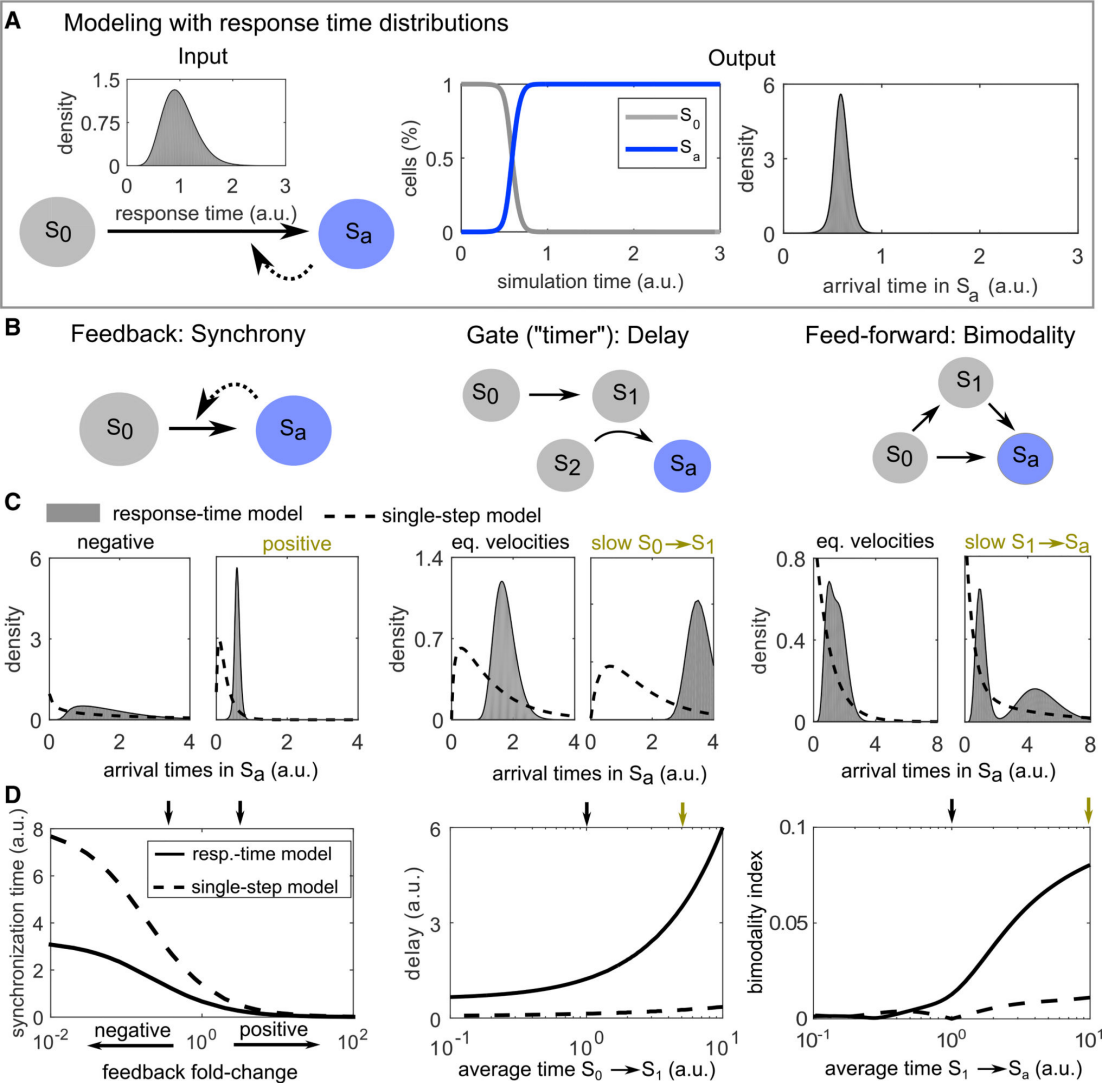
Network Motif Analysis Using Response-Time Modeling (A) Illustration of response-time modeling: each reaction arrow represents an intracellular multi-step process represented by a gamma distribution *γ*(*α, β; t*) (Equation 1). The process is started in state *S*_0_ and continues until all cells reach the absorbing state *S_a_*. Dashed arrow: positive feedback. Arrival time: *a posteriori* distribution of the times to reach state *S_a_* considering feedback. (B) Simple models (network motifs) of cell-to-cell communication. (C and D) Simulations of the models shown in (B). To keep the response-time models and single-step models comparable, we scaled the rate parameter of the gamma distributions as *β*→*αβ*, so that the average of the distribution is 1/*β* independently of *α*. Feedback and interaction (gate motif) are modeled by Michaelis-Menten type equations (see STAR Methods). Parameter values used in (C) are indicated by small color-coded arrows in (D). “Average time”: Average 1/*β^base^* of the gamma distribution representing the respective reaction. “eq. velocities”: equal average times for both reactions. Parameters not stated otherwise: *α* = 10 (“response-time model”) or *α* = 1 (“single-step model,” i.e., the exponential distribution is used), *K* = 0.1, *β^base^* = 1, feedback fold-change *η* = 5 (positive feedback) and 0.2 (negative feedback). In the feedforward loop motif, the branching probability is *p*_01_ = *p*_02_ = 0.5.

Having established the typical response-time patterns emerging from intracellular processes, we next asked how more general cell-state transitions shape dynamic response patterns of cell populations. For this purpose, we made use of response-time modeling (Figure 3A), which describes a cell-state change from a state *S_i_* to *S_j_* at time *t*, when starting at time *τ*, by a response-time distribution *ψ_ij_*(*t* − *τ*). We provide an introduction to response-time modeling with definitions and technical references (Box 1), and a case study on IL-2 competition between Th and regulatory T cells (Box 2).

In our implementation of response-time modeling, we specifically chose gamma-distributed response times (Equation 1) because of their frequent occurrence in intracellular processes (Table 1; Figures 2B–2E). An advantage of this approach is that we can consider cell-to-cell interactions including feedback (e.g., by exchange of diffusible ligands) simply as a dependence of the parameters of the gamma distribution on the fraction of cells in a certain cellular state *S_l_* (Box 1):

(Equation 2) $$\psi_{\text{ij}}(S_{l}(t),t-\tau )=\gamma (\alpha_{\text{ij}}(S_{l}),\beta_{\text{ij}}(S_{l});t-\tau ).$$

To completely determine the system, one needs to also provide probabilities *p_ij_* for the execution of each possible reaction (with Σ_*i*_*p_ij_* = 1), e.g., in the case of branching reactions. Note that, in the basic framework presented here (Equation 2), we assume a “well-stirred” situation and do not take into account spatial effects like concentration gradients in diffusible messengers (Thurley et al., 2015).

Using response-time modeling, we analyzed a set of simple toy models or “network motifs” that often appear in larger cell-to-cell communication networks (Figure 3B). In analogy to the simple multi-step models studied in Figure 2, our main readout is the *a posteriori* distribution of arrival times in an absorbing state *S_a_* (Figure 3C) (in general, every state of interest can be treated as an absorbing state by removing transitions leaving that state [Van Kampen, 2002]). In contrast to the response-time distributions from simple models shown in Figures 2A–2E, the arrival-time distributions are not normalized, but are analyzed separately within each motif to reveal the effect of parameter values (see STAR Methods).

As a first example, consider a single cell-state transition with feedback (Figures 3B–3D, “feedback”). We found that positive feedback decreases and negative feedback increases the width of the arrival-time distribution. In our model, that effect is stronger for feedback between cells than for intracellular feedback (Figures S2A and S2B). Intuitively, this happens in this simple model because intercellular feedback immediately and globally activates all cells, whereas intracellular feedback acts just within single cells and has no “snowballing” effect across the population. For quantification, we defined the “synchronization time” as the minimal time frame in which a certain fraction of cells (here 75%) responds after an initial delay time (see STAR Methods). Feedback between cells has only minor effects on this delay time (Figures S2C and S3A), but has strong effects on the synchronization time (Figures 3D and S2C). Thus, feedback regulation between cells is well suited to generate highly synchronized or desynchronized responses across a cell population.

Conversely, we asked whether a simple cellular communication network could control the delay without changing synchronization—a sort of “timer” circuit for the cellular population. Indeed, we found that long delays can be achieved without increased synchronization times by adding a bottleneck, e.g., in terms of the positive interaction or “gate” motif (Figures 3B–3D, “gate”). The gate motif increases delay without changing synchronization over a wide parameter range (Figures 3D and S3A). Adding delay by simply slowing down the intracellular processes that induce one or two consecutive cell-state changes is not sufficient for this effect, as here the synchronization time increases substantially when adding delay (Figures S3B–S3D). Intuitively, the higher level of synchronization in the gate motif can be explained by the global positive interaction, which increases synchronization similar to the positive feedback case, and therefore compensates for the loss of synchronization.

Finally, we studied the redundant, coherent feedforward loop. This motif is a simple model of the situation that cellular activation (reaching state *S_a_*) can be induced in several different ways, for example by means of different types of cytokines. We found that this motif can generate a bimodal distribution of arrival times in the absorbing state (Figures 3B–3D, “feedforward”). Intuitively, that bimodality is caused by the contributions from the “direct” and the “indirect” (via *S*_1_) ways to reach *S_a_*. However, substantial bimodality only arises if there is a timescale separation between the two routes to *S_a_*, as implemented here by a longer average time in the process *S*_1_→*S_a_*; otherwise there is no clear separation between the two peaks (Figures 3C and 3D), giving rise to a single long-lasting cellular response of moderate intensity (i.e., larger synchronization time, see Figure S3A). While bimodal distributions can also occur by crosstalk inside intracellular networks (essentially also a feedforward loop) (Figure 2E), a feedforward loop of elementary reactions is not sufficient for bimodality (Figures 3C and 3D, dashed lines, and STAR Methods).

### Cell-to-Cell Communication Allows Independent Control of Delay and Synchronization

Above, we used the prototypic response-time distributions arising in intracellular signal transduction networks to analyze common network motifs of cell-state dynamics. We found that, in the framework of response-time modeling, simple network motifs can induce emergent behavior such as bimodal response times, which does not arise in the corresponding single-step models that neglect the multi-step nature of cell-state changes.

**Figure 4:**
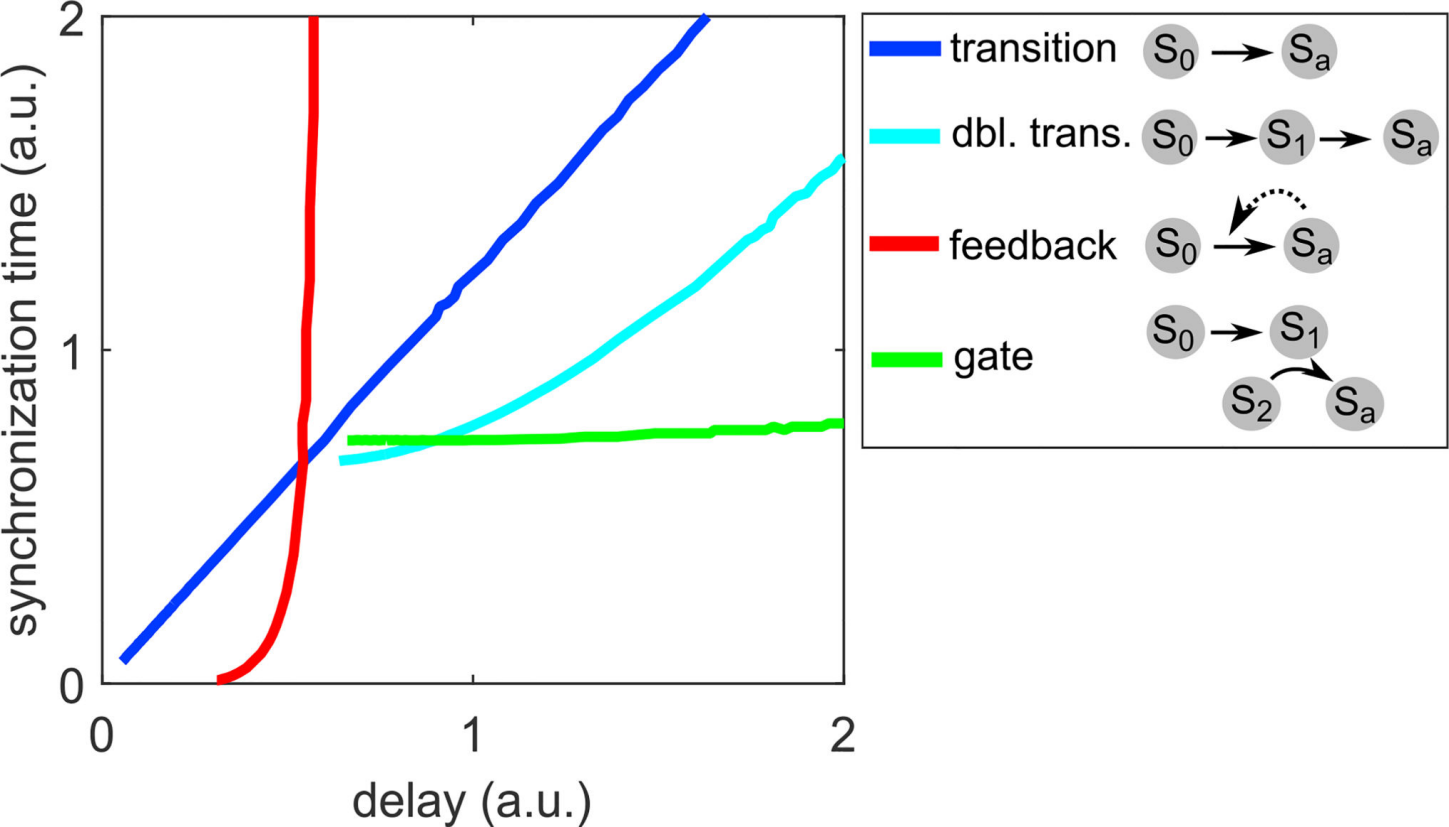
Independent Control of Delay and Synchronization Comparison of the gate and feedback motifs (Figures 3B–3D), and the single-or double-state transition motifs (Figures S3B–S3D). The gate motif allows for variable delays for the same degree of synchronization while, conversely, the feedback motif allows variable synchronization for the same delay. Synchronization and delay cannot be decoupled for the single transition model, which represents intracellular multi-step processes alone (see Figures 2A–2D). All curves are generated by changing the timescale or feedback strength parameters as in Figures 3D and S3D.

In addition, our simulations revealed important roles of cell-to-cell interaction circuits in shaping signaling dynamics. We found that simple circuits, such as the feedback or gate motif, can control synchronization and delay independently (Figure 4). In contrast, for intracellular multi-step processes described by a gamma distribution, synchronization and delay were linearly related and could not be controlled separately (Figure 4, blue line).

### Delay-Induced Persistence Detection

**Figure 5:**
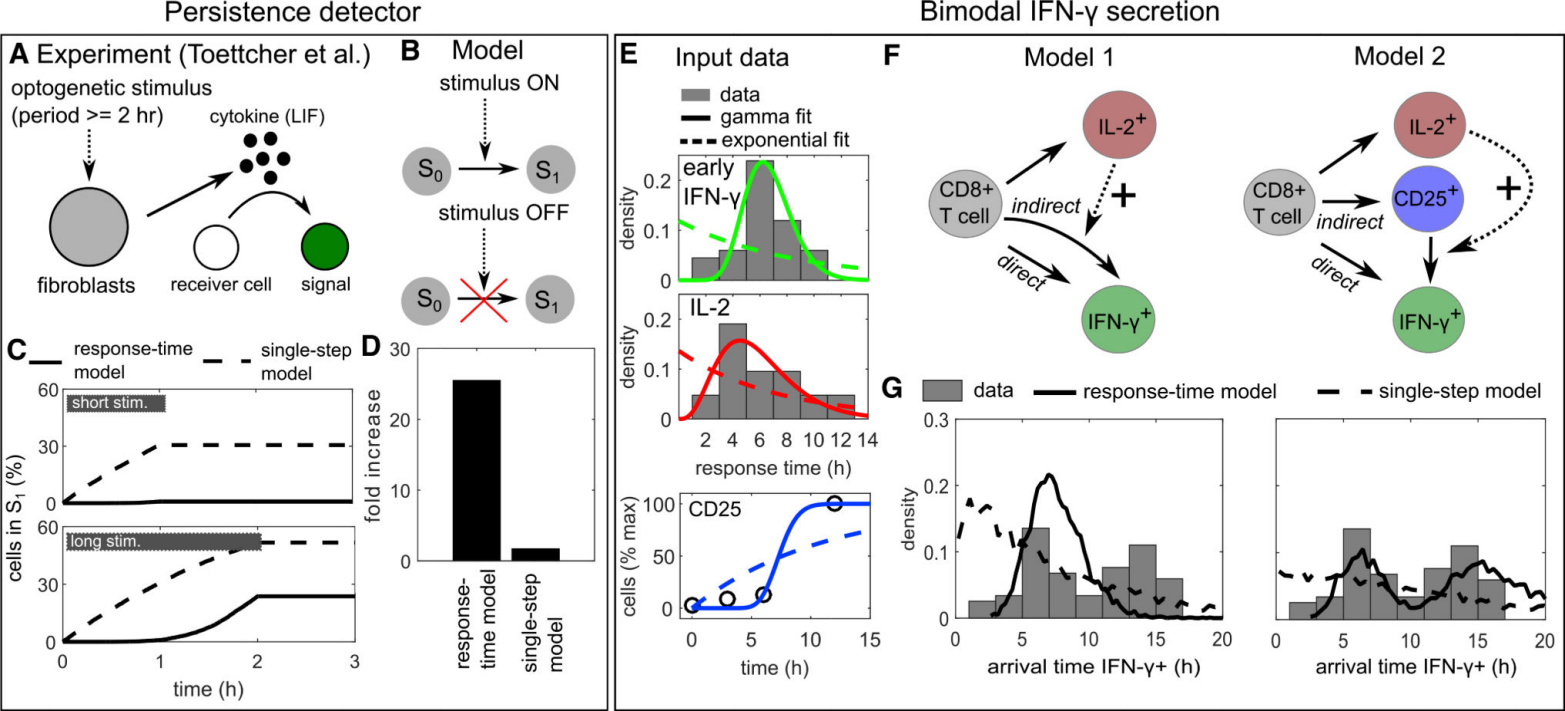
Persistence Detection and Bimodal IFN-γ Secretion Onset (A) Persistence detection: in the experiment by Toettcher et al. (2013), receiver cells showed a fluorescent signal (state-change) when exposed to an opto-genetic stimulus for 2 hr but not for 1 hr; a paracrine cytokine signal was essential for the effect. (B) Model schema: a stimulus triggers a cell-state change that is governed by a response-time distribution. The reaction is stopped when the stimulus terminates. (C and D) Simulation of the model in (B) with the best-fit gamma distribution (“response-time model”) or the best-fit exponential distribution (“single-step model”) derived from measured IL-2 secretion onset times (Figure 1A; Table 1). (C) Kinetic simulations. Gray bars indicate stimulus duration. (D) Fold increase: relative increase in maximal activity from short to long stimulus. (E) IFN-γ secretion onset: input data used for the models (see also Table S1). IL-2 and IFN-γ secretion onset times were taken from Han et al. (2012) (Figure 1A), and the initial IFN-γ secretion onset times were obtained by cutting after the dip at 10 hr and renormalizing. Kinetics of CD25 (α subunit of IL-2R) upregulation were taken from Dorner et al. (2009) and normalized to maximal expression. Fitting lines show best-fit curves to gamma and exponential distributions (for CD25, the corresponding cumulative distribution function was used). (F) Response-time models of IL-2 and IFN-γ secretion onset. Solid arrows represent an intracellular multi-step process represented by a response-time distribution and a probability to execute each of the branching reactions (see Table S1). Dashed arrows represent positive interaction. (G) Simulation of the models in (F): *a posteriori* arrival times to reach state “IFN-γ+,” i.e., initiate IFN-γ secretion. Note that the probabilities *p_ij_* for each differentiation path (IL-2+, CD25+, and IFN-γ+) are determined independently of the response-time distributions (see Table S1), and therefore the shown arrival times are normalized to the corresponding fraction of cells: given a certain differentiation path, it is certain that the new state will be reached eventually. “Response-time model”: simulations with best-fit gamma distributions (here non-integer valued shape parameters are possible); “single-step model”: simulations with best-fit exponential distributions.

A network that rejects transient activation signals and only responds to persistent signals has been termed a “persistence detector” (Mangan et al., 2003; Shen-Orr et al., 2002). Persistence detection in cell-to-cell communication has recently been demonstrated in the context of a paracrine signal induced by opto-genetic tools, which can precisely control the timing of an input stimulus (Toettcher et al., 2013) (Figure 5A). In that experiment, a 2-hr, but not a 1-hr stimulus, causes the cell-state change of a silent “receiver cell” to a cell with detectable fluorescence signal. Toettcher et al. (2013) report that this cell-state change is mediated by a paracrine cytokine signal (the IL-6 family cytokine leukemia inhibitory factor), but the precise mechanism of persistence detection in that system is still to be resolved. Following our observation that cytokine-secretion onset often shows considerable delays with gamma-distributed onset times (Table 1), we wondered whether such delays are sufficient to explain the persistence detection reported by Toettcher et al. (2013). To study that question, we analyzed a simple model (Figure 5B) where cell-state transitions are executed with the response-time distribution measured for IL-2, and are triggered by an external stimulus that is present for either 1 or 2 hr. Indeed, we found that the response-time model, but not the single-step model, yields a strong difference in cell activation between the 1- and 2-hr stimuli (Figures 5C and 5D). Thus, delays in cytokine secretion onset can indeed explain the reported persistence detection.

For a more generic analysis, we started from our finding that signaling circuits, such as the gate motif, can control delay independently of synchronization (Figure 4). We again assumed an input stimulus triggering a cell-state change (see Figure 5B), now acting on a cell-state change controlled by an underlying “delay-inducing” gate or transition network motif. We modeled these motifs using our response-time approach (i.e., fit gamma distributions to their input-to-output relationship) and scaled the average response times for both motifs, so that they have the same delay (3 time units). Our simulations show that both delay-inducing motifs exhibit some degree of persistence detection compared with a simple, single-step process (Figure S4A), but only the gate motif allows for 100% of the cells to be activated for a long stimulus (e.g., 5 time units) while still rejecting a short stimulus (e.g., 3 time units). Moreover, the gate motif has a sharp transition in signal amplitude for varying stimulus duration or delay time (Figure S4B).

In summary, effective persistence detection requires both substantial synchronization and a delay longer than the stimulus duration (Figure S4B). Both conditions are fulfilled for reported cytokine secretion onset times (Figures 5C and 5D), which therefore might have a functional role in rejecting transient input signals to interacting cell populations. Future experimental research will show to what extent that mechanism of persistence detection applies to the system studied by (Toettcher et al., 2013) and other interacting cell populations.

### Bimodal IFN-γ Secretion Onset Times

In our literature survey (Table 1), an example of a response-time distribution that clearly deviates from the commonly observed single-peaked pattern is the bimodal IFN-γ secretion onset times (Han et al., 2012) (Figure 1A). Our analysis of intercellular communication networks suggested that a feedforward loop motif can evoke a bimodal response-time distribution (Figures 3B–3D, feedforward). As it is known that IL-2 stimulates IFN-γ secretion of CD8+ T cells (Kasahara et al., 1983; McDyer et al., 2002), we next examined whether a combination of direct (antigen driven) and indirect (IL-2-mediated) stimulation of IFN-γ secretion is sufficient to explain the bimodal distribution.

Response-time modeling allows annotating cell-state models by directly using measured transition probabilities and response-time distributions. In that way, we were able to completely specify the process (except for the IL-2 interaction strength, see Figure S4C) based on a published dataset (Han et al., 2012) (Figure 5E; Table S1): The onset times of IL-2 secretion are well described by a gamma distribution, and the same is true for the early IFN-γ onset times. For late (indirect) IFN-γ secretion, we used the same distribution modified by IL-2 interaction (Figure 5F, model 1) (STAR Methods). The reasoning was that likely similar pathways are involved in the production and secretion of IFN-γ in both the direct and indirect case, but that they are activated either directly by antigen or indirectly via IL-2 (possibly after weak antigenic pre-stimulation). To simulate the process, we used a generalized Gillespie algorithm (Boguñá et al., 2014), which is necessary here because some of the input gamma distributions have a small non-integer valued shape parameter (see STAR Methods).

Clearly, the response-time distribution generated by model 1 is not bimodal, and does not explain the data even qualitatively (Figure 5G). The reason is that the initial onset time distributions for IL-2 and IFN-γ are too similar, and therefore their combination leads to a single broad peak rather than a second peak in the response times (cf. Figure 3C, feedforward loop). Thus, we reasoned that another mechanism must account for this observed delay. In fact, unstimulated T cells express only very limited amounts of the high-affinity IL-2 receptor CD25, and therefore we asked whether stimulation-induced CD25 upregulation may cause that additional delay (Figure 5F, model 2). For this process, we used CD25 expression kinetics of CD8+ T cells measured in (Dorner et al., 2009), which are also well described by a gamma distribution (Figure 5E). Indeed, model 2 generates a bimodal distribution for IFN-γ secretion onset, and is in good qualitative agreement with the reported values (Figure 5G). The IL-2 induction is not strictly necessary for a bimodal distribution and merely shifts probability mass to the left peak (Figure S4C)—thus, the model results do not qualitatively change over a substantial range of the only free parameter, the IL-2 interaction strength. In contrast, the corresponding single-step model (dashed line) cannot reproduce the bimodal shape of the distribution. That demonstrates that our approach using the full response-time distribution is necessary to explain the data, and cannot be replaced by single-step models using only average response times.

## DISCUSSION

Our approach focuses on cell-to-cell communication and builds on the observation that intracellular cascades are often well described by gamma distributions (Table 1; Figure 2). We apply response-time distributions to model cell-to-cell communication networks, distinguish properties of simple network motifs, and apply this modeling approach to experimental data already in the literature to make testable predictions. Our goal with this approach is to facilitate the analysis of large and complex cell-to-cell communication networks.

Previous modeling studies of cell-to-cell communication networks have either represented cell-state changes using single-step processes, thus treating a cell-state change as if it was caused by a single molecular reaction (Gupta et al., 2011; Hart et al., 2012; Mathew et al., 2014; Sontag, 2017), or they have focused on specific cases and modeled the intracellular dynamics in some detail (Feinerman et al., 2010; Fuhrmann et al., 2016; Schulz et al., 2009; Tay et al., 2010; Thurley et al., 2015; Youk and Lim, 2014). Response-time distributions have been explored earlier for model reduction techniques (Chevalier and El-Samad, 2009; Mastny et al., 2007) and to analyze specific biological systems (Callard and Hodgkin, 2007; Chevalier and El-Samad, 2014; Duffy et al., 2012; Hawkins et al., 2007; Helmstetter et al., 2015; Mittler et al., 1998; Paszek et al., 2010; Pedraza and Paulsson, 2008; Zilman et al., 2010), applications including gene expression dynamics, viral infection, T cell population dynamics, and noise propagation in signaling pathways. Those studies demonstrate that many complex biological systems cannot be adequately described by single-step-rate equation models, or at least not in their physiological environment.

The challenge in detailed modeling of cellular dynamics is not only that biological networks are incompletely understood, so that pathway maps are incomplete. Rather, the fundamental problem is that the parameters needed to describe cellular networks (e.g., reaction rate parameters for all subprocesses involved in expression of a gene) cannot all be determined *in vivo*. In contrast, response-time distributions and branching probabilities can be measured with high temporal accuracy via current single-cell technologies, such as multi-color fluorescence-activated cell sorting or mass cytometry, live-cell imaging, and RNA sequencing (Polonsky et al., 2016; Shalek et al., 2014; Spitzer and Nolan, 2016) (see Table 1).

Here, we explored a recursive implementation of response-time modeling as a framework for mathematical analysis of cell-to-cell communication networks (Figure 1). This approach relies on the finding that intracellular dynamics often evoke single-peaked response-time distributions (Figure 2). Analyzing simple network motifs, our approach revealed dynamical properties such as bimodal arrival times and enhanced synchronization, which are masked when treating cell-state changes as molecular reactions (Figures 3 and 4). Finally, we demonstrated that our framework can be used to analyze currently available experimental data on cytokine secretion onset times (Figure 5). We note that, in a mathematical sense, it is somewhat arbitrary which parts of a network are isolated and summarized as “subnetworks” for a response-time modeling approach. In fact, compartments inside a cell might be regarded as interacting subunits, or several cells in a certain microenvironment might form a community that interacts with other communities; such cases may, in principle, be treated analogously in the framework of response-time modeling.

The analysis of common network motifs has a long tradition in systems biology, and was used to elucidate metabolic networks (Heinrich et al., 1977) and gene-regulatory networks (Hornung and Barkai, 2008; Ma et al., 2009; Shen-Orr et al., 2002), among others. The reasoning is that large, physiologic networks are composed of small, functional network motifs and can be rationalized based on these building blocks. To demonstrate such an approach for cell-to-cell signaling, we elucidated two published examples of intercellular interaction. We found (1) that the paracrine persistence detector (Toettcher et al., 2013) can be explained by a delayed response-time distribution, which possibly stems from the onset of cytokine secretion, and (2) our analysis of IFN-γ secretion onset times (Han et al., 2012) revealed that the observed secondary response can be explained by a feedforward loop motif consisting of IL-2 secretion and IL-2 receptor upregulation. Those results provide a rationale for plausible mechanisms that can be tested experimentally in future research. Moreover, both examples demonstrate an advantage of our modeling approach, which is that no or very few free parameters need to be assigned if the response-time distributions of key processes are measured directly.

Cell-to-cell interaction is crucial for many functions of higher organisms, and complex intercellular communication networks have been discovered over the last decades. While the experimental capabilities to elucidate cellular responses to specific input stimuli are becoming increasingly available—sometimes even for spatiotemporal, single-cell analysis (Polonsky et al., 2016)—there will always be missing information. Response-time modeling offers a timely approach for predicting communication network structure and behavior using experimentally accessible input-to-output measurements even without detailed knowledge of intermediate steps.

## STAR★METHODS

Detailed methods are provided in the online version of this paper and include the following:

- KEY RESOURCES TABLE
- CONTACT FOR REAGENT AND RESOURCE SHARING
- METHOD DETAILS
  ○ Simple Models of Intracellular Processes
  ○ Implementation of Response-Time Models
  ○ Generalized Gillespie Algorithm
  ○ Formal Calculations
- QUANTIFICATION AND STATISTICAL ANALYSIS
  ○ Measures of Response-Time Distributions
- DATA AND SOFTWARE AVAILABILITY

## STAR★METHODS

### CONTACT FOR REAGENT AND RESOURCE SHARING

Further information and requests for resources and reagents should be directed to and will be fulfilled by the Lead Contact, Kevin Thurley (kevin.thurleydrfz.de).

### METHOD DETAILS

#### Simple Models of Intracellular Processes

##### General Approach

The model schemes in Figures 2B–2E were translated into differential rate equations by standard methods (see section Model Equations below). In all models with a single absorbing state *x_n_* (except the parallel chain) and without cellular heterogeneity in the reaction rate parameter, the response-time (or first-passage time) distribution can be obtained directly from the differential equation solutions as the flux into the absorbing state *x_n_* (Van Kampen, 2002): $\psi_{n}(t)=\frac{{\text{dx}}_{n}}{\text{dt}}$. In the case of the irreversible chain, the response-time distribution has a closed-from expression, the gamma distribution $\gamma (\alpha ,\beta ;t)=\frac{t^{\alpha -1}e^{-\beta t}\beta^{\alpha }}{\Gamma (\alpha )}$, where *Γ*(*α*) is the Euler gamma function. In that parameterization of the gamma distribution, *α* may be interpreted as the “non-integer valued step number”, and then is the reaction rate of each step (see Equation 1). For the parallel chain, the probability to reach the final state *n* in the first-out-of-*m* parallel processes, $f_{m}(t)=m\psi_{n}(t){\left(1-\int_{0}^{t}\psi_{n}(t\prime )\text{dt}\prime \right)}^{m-1}$ (Lu et al., 2006), is taken as the response-time distribution.

To account for cellular heterogeneity, we replaced the uniform rate parameter *λ* by a log-normal distributed rate parameter *λ*, i.e. *λ* was drawn from a log-normal distribution $g(\lambda )=\frac{1}{s\lambda \sqrt{2\pi }}\text{exp}[-\frac{{\left(\text{ln}(\lambda )-m\right)}^{2}}{2s^{2}}]$ for each cell. The parameters *s* and *m* are chosen as to have a coefficient of variation CV (standard deviation/average) as indicated. Subsequently, the response-time distribution is obtained by stochastic simulation (*n* = 20000) using Gillespie’s algorithm.

##### Model Equations

For all models (except the cascade model), we impose the normalization condition $\sum_{i=0}^{n}x_{i}=1$ and the initial condition *x*_0_(0) = 1 and *x_i_*(0) = 0, for *i* > 0.

###### a) Single step process

Single irreversible, molecular reaction.

Master equation: $\frac{{\text{dx}}_{1}}{\text{dt}}=\lambda x_{0}$.

Response-time distribution: *ψ*_1_(*t*) = *λe*^−*λt*^, exponential distribution.

###### b) Irreversible chain

A chain of n molecular reactions.

Master equation: $\frac{{\text{dx}}_{i}}{\text{dt}}=\frac{\lambda }{n}(x_{i-1}-x_{i})$, *i* = 1…*n* − 1; $\frac{{\text{dx}}_{n}}{\text{dt}}=\frac{\lambda }{n}x_{n-1}$.

Response-time distribution: $\psi_{n}(t)=\frac{t^{n-1}e^{-\lambda t}\lambda^{n}}{\Gamma (n)}$, i.e. the gamma distribution.

###### c) Parallel irreversible chain

Here, we assume that the active state of a cell can be reached by any of *m* molecules transitioning to the active state through a multi-step process.

Response-time distribution (Lu et al., 2006): *ψ_n,m_*(*t*) = *mψ*_*n*,1_(*t*)(1 − ∫ *ψ*_*n*,1_(*t*)*dt*)^*m*−1^

where *ψ*_*n*,1_(*t*) = *ψ_n_*(*t*) in the irreversible chain. In words, *ψ_n,m_*(*t*) is the probability density to reach state *n* for the first time at *t*, and can be computed as *m* times the probability for a single molecule to reach the active state, given that it has not been reached earlier by any of the other *m*−1 molecules.

###### d) Reversible chain

Here instead of Equation 1, we consider reversible reactions.

Master equation: $\frac{{\text{dx}}_{i}}{\text{dt}}=\frac{\lambda }{n}(x_{i-1}-x_{i})+\frac{k}{n}(x_{i+1}-x_{i})$, *i* = 1…*n* − 1, *k* > *λ*.

Response-time distributions are computed numerically from the master equation.

###### e) Cascade

We adopted the model from Heinrich et al. (Heinrich et al., 2002).

Master equation: $\frac{{\text{dx}}_{i}}{\text{dt}}=\lambda (1-x_{i})x_{i-1}$, *i* = 1…*n*, and *x*_0_(*t*) = 1 for all *t* > 0 (an activated receptor). Here, *x_i_*(*t*) is the probability that the i-th kinase is active, and therefore the *x_i_*(*t*) are separately normalized and take values in the interval [0,1]. Initial conditions: *x_i_*(0) = 0, *i* = 1…*n*.

Response-time distributions are computed numerically from the master equation.

###### f) Cross-talk

Similar to irreversible chain, but with a “short-cut” reaction from *x_l_* to *x_n_*.

Master equation: $\frac{{\text{dx}}_{i}}{\text{dt}}=\frac{\lambda }{n}(x_{i-1}-x_{i})$, *i* = 1…*n* − 1, *i* ≠ *l*; $\frac{{\text{dx}}_{l}}{\text{dt}}=\frac{\lambda }{n}(x_{l-1}-x_{l})-\lambda x_{l}$;

$$\frac{{\text{dx}}_{n}}{\text{dt}}=\frac{\lambda }{n}x_{n-1}+\lambda x_{l}.$$

Response-time distributions are computed numerically from the master equation.

#### Implementation of Response-Time Models

##### Network Motifs

All models shown in Figures 3 and 4 are implemented using a “linear chain” framework (Box 1). That means the models depicted in Figures 3B and S3B each result in a system of ordinary differential equations. Here, feedback and interaction are modelled by a dependence of the rate parameter *β* of the input gamma distribution to the fraction of cells in a state *S_l_* (Equation 2, Box 1). For positive and negative feedback (Figures 3B–3D, “feedback”, and Figure S2), we used $\beta (S_{l})=\beta^{\text{base}}\frac{K+\eta S_{l}}{K+S_{l}}$ in Equation 2, where *β^base^* is the base-level rate parameter, and the fold-change *η* determines feedback type and strength (positive feedback: *η* > 1, negative feedback: *η* < 1). For cellular interaction (Figures 3B–3D, “gate”; Figure 5E), we used $\beta (S_{l})=\beta^{\text{base}}\frac{S_{l}}{K+S_{l}}$.

##### Persistence Detector Model

The persistence detector model (Figure 5A and B) can be expressed in the linear-chain formulation as follows:

$$\frac{{\text{dS}}_{0}}{\text{dt}}=-\lambda f(t)S_{0}$$

$$\frac{{\text{dx}}_{k}}{\text{dt}}=\lambda f(t)(x_{k-1}-x_{k}),\text{ for }k=2\ldots n;$$

$$\frac{{\text{dS}}_{1}}{\text{dt}}=\lambda f(t)x_{n}$$

where *x*_1_ = *S*_0_, $f(t)=\{\begin{array}{cc} 1, & t<t_{d}\\ 0, & \text{otherwise} \end{array}\}$, and *t_d_* is the stimulus duration. The above equations are used in Figures S4A and S4B. In Figures 5C and 5D, the corresponding gamma distribution formulation is used with the gamma distribution parameters representing IL-2 secretion (Table 1). In that case, the model is implemented by step-wise, direct integration of the gamma distribution over the period of time where the stimulus *f*(*t*) is present.

##### Bimodal IFN-γ Secretion Model

The model (Figure 5E) implements the generalized reactions shown using response-time distributions, and in addition degradation of the IL-2 and IFN-γ secreting cell populations is considered, as described in the main text. The branching reaction (differentiation of CD8+ T cells towards IL-2+ cells or IFN-γ+ cells, directly or indirectly) is realized by assigning separate pools of CD8+ T cells in the initial conditions, according to the ratios experimentally observed by (Han et al., 2012) (see Table S1). The only free parameter is the strength of the IL-2 dependence on response-time for differentiation towards IFN-γ+ cells. Simulations are run using the generalized Gillespie algorithm described below, with *n* = 5000 cells.

##### IL-2 Competition Model

In the case study described in Box 2, response-time distributions for Th and Treg cell activation (i.e. CD25 up-regulation) are generated using the IL-2 receptor model from (Busse et al., 2010; Thurley et al., 2015) without IL-2 secretion, and at a given extracellular IL-2 concentration (Box Figure A, and Section “IL-2 receptor model” below). From that model, we use the kinetics of IL-2/IL-2 receptor complexes as a read-out (Figure S5A), which corresponds to the experimentally accessible parameter CD25 up-regulation (Busse et al., 2010; Thurley et al., 2015). That output is normalized after neglecting the initial steep increase to a basal level (1^st^ hr), so that the final curves reflect the kinetics of receptor up-regulation (Box Figure B). We identify that curve with the response-time distribution for T cell activation (i.e. IL-2 receptor up-regulation) and use best-fit gamma distributions to proceed (dashed lines Box Figure B), similar to the approach taken with the experimentally obtained response-time distributions (Figure 5E). Such response-time distributions are generated for both Th and Treg cells under a range of extracellular IL-2 concentrations (Figure S5B). The resulting α and β parameters of the gamma distributions (see Equation 1) for varying IL-2 concentrations are used to fit interpolating functions (dashed lines in Figure S5B): *α*([*IL* − 2]) = *C_α_* + (*K_α_*/[*IL* − 2])^*n*^ and *β*([*IL* − 2]) = *V_β_*[*IL* − 2]/(*K_β_* + [*IL* − 2]), each separately for Th and Treg cells. Taken together, the fitting parameters *C_α_, K_α_, n, V_β_, K_β_* completely describe the response-time distributions for Th cell and Treg cell activation in dependence of the extracellular IL-2 concentration.

To close the response-time model, it remains to determine the concentration of extracellular IL-2 depending on the degree of Th and Treg cell activation. A rigorous discussion of that question requires solving a non-linear diffusion problem in 3 spatial dimensions (Oyler-yaniv et al., 2017; Thurley et al., 2015). In a simplified scenario at steady state, the IL-2 concentration in the vicinity of a single cell can be calculated as the ratio of the production rate and IL-2 consumption by both diffusive escape and absorption by IL-2 receptors (Thurley et al., 2015): [*IL* − 2] = *q*/[*k*_on_*R* + 4*πD*ρ], where *q* is the IL-2 secretion rate, *k*_on_ is the IL-2 receptor binding rate, *D* = 10 µ*m*^2^/*s* is the diffusion constant, and ρ = 5 µ*m* is the typical cell diameter. Summing over all available IL-2 secreting cells and IL-2 receptors, and switching from absolute cell numbers to fractions *f*_Th_ (Th cells) and *f*_Treg_ (Treg cells) of the total cell density *σ*, we obtain: $[\text{IL}-2]=\frac{q\sigma f_{\text{Th}}}{k_{\text{on}}(R_{\text{Th}}\sigma f_{\text{Th}}A_{\text{Th}}+R_{\text{Treg}}\sigma f_{\text{Treg}}A_{\text{Treg}})+4\pi D\rho /\text{Vol}}$ where *A*_Th_, *A*_Treg_ is the current fraction of active cells among the Th or Treg cells, *R*_Th_, *R*_Treg_ is the total numbers of IL-2 receptors per Th or Treg cell (here: *R*_Th_ = 1000 molecules, *R*_Treg_ = 2000 molecules), and we consider a fixed diffusive volume *Vol* = 1 µl. The equation above calculates the extracellular IL-2 concentration based on the fraction of already activated Th and Treg cells and thus closes the response-time model.

In Box 2, the described model is simulated using the generalized Gillespie algorithm (see below) for IL-2 receptor dynamics, with *n* = 2000 cells (Th and Treg cells combined).

##### IL-2 Receptor Model

In Box 2 and Figure S5, we refer to the IL-2 receptor model from (Busse et al., 2010; Thurley et al., 2015). Here we re-print the equations for convenience of the reader (see Figure B1A in Box 2 for a model scheme and brief description):

$$\frac{\text{dR}}{\text{dt}}=v(C)-(k_{\text{on}}\text{RI}+k_{\text{iR}})R+k_{\text{off}}C+k_{\text{rec}}E$$

$$\frac{\text{dC}}{\text{dt}}=k_{\text{on}}\text{RI}-(k_{\text{off}}+k_{\text{iC}})C$$

$$\frac{\text{dE}}{\text{dt}}=k_{\text{iC}}C-(k_{\text{rec}}+k_{\text{deg}})E$$

$$v(C)=v_{0}+v_{1}\frac{C^{3}}{K^{3}+C^{3}}$$

Parameter values are as in the original publication: *k*_on_ = 112 nM^−1^h^−1^, *k*_iR_ = 0.64 h^−1^, *k*_off_ = 0.83 h^−1^, *k*_rec_ = 9 h^−1^, *k*_iC_ = 1.7 h^−1^, *k*_deg_ = 5 *h*^−1^, *K* = 1000 molecules/cell; *v*_0_ = 150 molecules/[cell h], *v*_1_ = 3000 molecules/[cell h] for Th cells, and *v*_0_ = 1000 molecules/[cell h], *v*_1_ = 8000 molecules/[cell h] for Treg cells.

#### Generalized Gillespie Algorithm

In the models describing bimodal IFN-γ secretion and competition for IL-2, we used a recently published algorithm (Boguñá et al., 2014), which efficiently simulates semi-Markov processes using an approximation that is valid for large numbers of cells. Indeed, our implementation shows excellent agreement with the exact solution available for a multi-step process when using > 1000 cells (Figures S4D and S4E). Essentially, the generalized Gillespie algorithm replaces the reaction rates γ_*i*_ used in the classic Gillespie algorithm by the hazard rates γ_*i*_(*τ*) = *ψ_i_*(*τ*)/*φ_i_*(*τ*), where *τ* is the elapsed time since the last reaction, *ψ_i_*(*t*) is the response-time distribution of process *i* and *φ_i_*(*t*) is the corresponding survival probability. In analogy to Gillespie’s algorithm, an “average rate” $\bar{\gamma }(\{t_{k}\})=N^{-1}\sum_{k=1}^{N}\gamma_{k}(t_{k})$ is computed (*N* is the number of active processes at a given reaction step), and the next reaction time and next reaction process are computed based on the expressions *N*γ̄({*t_k_*})*e*^−*N*γ̄({*t_k_*})*τ*^ and γ_*i*_(*t_i_*)/(*N*γ̄({*t_k_*})), respectively. A subtlety arises at time 0, where *t_i_* = 0 for all *i*, and therefore in the case of the gamma distribution, also γ_*i*_(*t_i_*) = 0 for all *i*. To avoid that case, whenever no active processes with *t_i_* > 0 are available (in particular at start), we used the exact implementation of the process (Equations 4 and 5 in (Boguñá et al., 2014)). In brief, a random time *τ* is drawn from the over-all survival probability $\theta (\tau |\{t_{k}\})=\prod_{k=1}^{N}\psi_{k}(\tau +t_{k})/\psi_{k}(t_{k})$ by solving *θ*(*τ*|{*t_k_*}) = *u* (*u* is a uniform random number), and a reaction channel *i* is chosen from the reaction probability $\gamma_{i}(t_{i}+\tau )/\sum_{k}\gamma_{k}(t_{k}+\tau )$, where γ_*i*_(*τ*) is defined as above. Here we implemented that exact stochastic simulation algorithm using an adaptive step-size *h* and repeatedly testing the condition *q*(*h* + … + *h*|{*t_k_*}) < *u*, until the next reaction time is found.

#### Formal Calculations

##### The Heterogeneous Single-Step Process Has a Monotonous Response-Time Distribution

In the main text, we claim that the heterogeneous single-step process never produces a peak in the response-time distribution; rather, monotonically decreasing, broad or long-tailed distributions arise, no matter how *λ* is distributed. This can be seen by the following calculation:

Consider two random variables, the time *t* until an event occurs, and the reaction rate *λ*, which is different in each cell. The joint probability distribution is given by *ψ_t,λ_*(*t,λ*) = *ψ*_*t*|*λ*_(*t*|*λ*)*ψ_λ_*(*λ*), where *ψ*_*t*|*λ*_(*t*|*λ*) is the response-time distribution conditioned on a fixed parameter *λ*. So in a single-step process, *ψ*_*t*|*λ*_(*t*|*λ*) = *λe*^−*λt*^, and the response-time distribution resulting from a stochastic parameter *λ* with distribution *g*(*λ*) is the marginal distribution *ψ_λ_*(*t*) = ∫ *ψ_t,λ_*(*t*|*λ*)*ψ_λ_*(*λ*)*dλ* = ∫ *λe*^−*λt*^*g*(*λ*)*dλ*. How does the distribution *g*(*λ*) effect the response time? With *g*(*λ*) > 0,

(Equation 3) $$\frac{d\psi_{\lambda }}{\text{dt}}=\int \lambda \frac{d}{\text{dt}}(e^{-\lambda t}g(\lambda ))d\lambda =-\int \lambda^{2}g(\lambda )e^{-\lambda t}d\lambda <0$$

Thus, for any *λ* > 0, *ψ_λ_*(*t*) always decreases monotonically and cannot have a peak, in contrast to the distributions arising from a multistep process.

##### Number of Peaks in Response-Time Distributions

In the main text, we claim that in the feed-forward loop motif (Figures 3B–3D, right panels), the *a posteriori* response-time distribution *ϕ*_02_(*t*) to reach the absorbing state *S_a_* = *S*_2_ when starting in *S*_0_ cannot be bi-modal (i.e. cannot have two maxima) when the state transitions are governed by exponential distributions. This can be seen by the following calculation:

The network of state transitions can be described by a Master equation (Van Kampen, 2002) for a vector ***S***(***t***) of the occupancy probabilities to be in state *S_i_* at time *t*:

(Equation 4) $$\frac{dS}{\text{dt}}=WS(t)$$

where the matrix element *W_ij_* = *λ_ij_* is the transition rate for a jump from *i* to *j*, and we impose initial conditions *S*_0_(0) = 1, *S_i_*(0) = 0, *i* = 1 … *n*.

For the feed-forward motif with transition rates *λ*_01_ = *a, λ*_12_ = *b, λ*_02_ = *c*, the matrix *W* reads

$$W=(\begin{array}{ccc} -a-c & 0 & 0\\ a & -b & 0\\ c & b & 0 \end{array}).$$

We next solve Equation 4. The first derivative of *S*_2_ is the *a posteriori* arrival time distribution, and the number of zeros of the second derivative informs us about the number of possible maxima. We consider two cases:

- For *b* ≠ *a* + *c, W* has three eigenvalues μ_*i*_ = (−*a*−*c*, −*b*, 0) and three independent eigenvectors, which form a basis for the solution of Equation 4. The solution is *S*_2_(*t*) = Σ*k_i_e*^−*μ_i_t*^ where $k_{1}=\frac{b-c}{a-b+c},\;k_{2}=\frac{-a}{a-b+c}$, *k*_3_ = 1. The derivative of the *a posteriori* arrival-time distribution, $\frac{d^{2}S_{2}}{{\text{dt}}^{2}}=k_{1}\mu_{1}^{2}e^{-\mu_{1}t}+k_{2}\mu_{2}^{2}e^{-\mu_{2}t}$, can have at most one zero, which happens in cases where *k*_1_, *k*_2_ have different signs (e.g. for *a* + *c* > *b* and *b* > *c*).
- In the degenerate case, *b* = *a* + *c* in Equation 4, the solution reads $\phi_{02}(t)=\frac{{\text{dS}}_{2}}{\text{dt}}=e^{-(a+c)t}[(a+c)(1+\text{at})-a]$, which has a single maximum at $t=\frac{c}{a(a+c)}$.

Thus, the feed-forward loop with elementary reaction steps only constrains the system to either a single or no peak.

### QUANTIFICATION AND STATISTICAL ANALYSIS

#### Measures of Response-Time Distributions

##### Delay

We defined the delay time *t*_delay_ as the longest time before ≤5% of a cell population reach the active state, so it is the 5-percentile of the response-time distribution.

##### Bimodality

To quantify bimodality, we used the standard error (root-mean square of the sum of residuals) of a best-fit to the gamma distribution, with the rational that a bimodal distribution cannot be fit by a single gamma distribution. This approach has been widely used with normal distributions (“dip-test” (Freeman and Dale, 2013)).

##### Synchronization

We define the synchronization time *t*_sync_ to a given arrival time distribution *ψ*(*t*) by the time elapsed after the delay time (see above) in which a certain fraction *d* of cells (here *d* = 75%) has responded for the first time. That time is given by the condition *P*(*t*_delay_, *t*_sync_) = *d*, where *P*(*t,τ*) = *F*(*t* + *τ*) − *F*(*t*)/[1 − *F*(*t*)] is the future life time, and *F*(*t*) is the cumulative probability distribution to *ψ*(*t*).

### DATA AND SOFTWARE AVAILABILITY

All computer simulations and analyses were carried out in Matlab R2015a. The code is available from the authors upon request.

## Supplementary Material

1

2

## Figures and Tables

**Table 1:** Literature Survey of Response-Time Distributions

Description	Average	CV	Distribution	References
Secretion onset of IL-2	6.3 hr	0.4	gamma (Figure 1A)	Han et al. (2012)
Secretion onset of TNF-α	3.6 hr	0.5	gamma (Figure 1A)	Han et al. (2012)
Secretion onset of IFN-γ	9.6 hr	0.4	bimodal (Figure 1A)	Han et al. (2012)
Production period IFN-γ (CD4+ T cells)	5.9 hr	0.61	gamma	Helmstetter et al. (2015)
Onset of IFN-β induction	3.3 hr	0.4	gamma	Rand et al. (2012)
IL-2 receptor upregulation	54 hr	0.35	gamma	Waysbort et al. (2013)
Transcription on times	5–20 min	1	exponential	Suter et al. (2011)
Transcription off times	0.5–3 hr	0.9	double-exponential	Suter et al. (2011)
Calcium interspike intervals in HEK cells	0.5–8 min	0.27	single-peaked	Thurley et al. (2014)
Calcium interpuff intervals in HEK cells	0.5–2 s	0.94	exponential or single-peaked	Thurley et al. (2011)
Lambda induction in bacteria (lysis)	100 min	0.13	single-peaked	Amir et al. (2007)
TLR4 endosome maturation time	4.4 hr	0.3	normal	Cheng et al. (2015)
Enzymatic reaction	10–50 ms	1–1.5	exponential or multi-exponential	English et al. (2006)
Cell-cycle time of retinal progenitor cells	56 hr	0.34	normal	Gomes et al. (2011)

Note that normal, gamma, and double- or multi-exponential distributions all fall into the class of “single-peaked” distributions. CV, coefficient of variation.

**Table:** KEY RESOURCES TABLE

REAGENT or RESOURCE	SOURCE	IDENTIFIER
Software and Algorithms		
Generalized Gillespie algorithm	This paper; Boguñá et al., 2014	N/A
Other		
Cytokine secretion kinetics in CD8+ T cells	Han et al., 2012	N/A
CD25 expression kinetics in CD8+ T cells	Dorner et al., 2009	N/A
Models for IL-2 receptor expression and IL-2 diffusion	This paper; Busse et al., 2010; Thurley et al., 2015	N/A

## Highlights

- Scalable modeling framework for modeling cell-to-cell communication networks
- Intracellular, input-to-output processing described by response-time distributions
- Emergent behaviors of cell-to-cell communication network motifs are identified
- Response-time modeling is applied to published cytokine secretion data

## Box 1 Response-Time Modeling

In cell-cell communication networks, cells receive signaling input from other cells. They process this information to potentially effect an internal state change, which in turn regulates when and which signals they output to affect the overall milieu of signals received by other cells. The approach we take is to replace complex, detailed models of intracellular signaling networks with probability distributions of cellular response times for changing states (see Box 2 for an instructive example). We model cell-cell communication by stochastic cell-state changes, and response-time distributions are dynamically updated based on cellular output signals (Figure 3A). The response-time distributions are also known as phase-type distributions in queueing or risk theory (Bladt, 2005) or as first-passage-time distributions in physics (Van Kampen, 2002). Moreover, response-time modeling can be formulated in terms of semi-Markov processes and a corresponding set of integral equations (generalized master equations) (Howard, 1971; Kenkre et al., 1973).

More formally, each cell can be in multiple states {*S_i_*}. The time between each direct state transition *S_i_*→*S_j_* is determined by a response-time distribution *ψ*(*j,t*|*i,τ*), the probability to arrive in state *j* for the first time at time *t* after having entered state *i* at time *τ*. In many cases, we can assume *ψ*(*j,t*|*i,τ*) = *ψ_ij_*(*t*−*τ*); i.e., the response times depend only on the time elapsed since the previous state change. To completely determine the process, we assign probabilities *p_ij_* for the occurrence of each state change *S_i_*→*S_j_* that can occur at a branching point, with the normalization Σ_*j*_*p_ij_* = 1 (Howard, 1971). Given that the cellular interaction network has an absorbing state *S_a_*, then the *a posteriori* arrival-time distribution *ϕ*_0*a*_(*t*) (arrival times in *S_a_* after starting in *S*_0_) can be computed as the flux into that absorbing state, $\phi_{0a}(t)=\frac{{\text{dS}}_{a}}{\text{dt}}$ (Van Kampen, 2002). Adding feedback or interaction may require an additional dependency on the number of cells in a certain state *S_l_* (see Equation 2 in the main text): *ψ_ij_* = *ψ_ij_*(*S_l_*(*t*),*t*−*τ*), which means that the integral equations describing the process become non-linear (Howard, 1971). Note that such feedback in the response times is different from delayed feedback (such as in a rate equation $\frac{{\text{dS}}_{i}}{\text{dt}}=F[S_{i},\int_{0}^{t}S_{l}(\tau )\psi_{n}(t-\tau )d\tau ]$, which has been widely studied in population biology; Smith, 2010).

Solving non-linear integral equations describing generalized state transitions is an analytically and numerically hard problem. However, a direct solution is possible in the case of gamma-distributed response times with integer-valued shape parameters. In this case, we can replace each response-time distribution by the corresponding irreversible *n*-step process, reducing the problem to an ODE system (see Equation 1 and Figure 2B). Specifically, we can replace each of the processes *S_i_*→*S_j_* by an *n*-step linear reaction chain

$$\frac{{\text{dS}}_{i}}{\text{dt}}=-\lambda S_{i}$$

$$\frac{{\text{dx}}_{k}}{\text{dt}}=\lambda (x_{k-1}-x_{k}),\;x_{1}=S_{i},\;k=2\ldots n$$

$$\frac{{\text{dS}}_{j}}{\text{dt}}=\lambda x_{n}$$

This procedure is a variant of the “linear chain trick” (Smith, 2010), and it naturally generalizes to systems with interaction or feedback in the rate parameter *λ*. In that case, we can write *λ* = *λ*(*S_l_*(*t*)), yielding a non-linear system of differential equations for *S_j_*(*t*).

## Box 2 Case Study of Response-Time Modeling for IL-2 Competition between Th and Treg Cells

Here, we examine a case study of an immunological cell-cell communication network in which previous studies revealed an interesting “competitio” mechanism among cells. (Busse et al., 2010; Feinerman et al., 2010; Fuhrmann et al., 2016; Oyler-yaniv et al., 2017; Thurley et al., 2015). In this cell-cell communication network, we consider (Figure B1A): nodes are helper T(Th) and regulatory T(Treg) cells; input is the cytokine IL-2, which comes from Th cells after antigenic stimulation; and the output is the cell state (inactive/active) as determined by IL-2 receptor (IL-2R) levels (down/up regulated; this is typically measured by the surface marker CD25). An interesting property of this particular cell-cell communication network is that IL-2R binds IL-2 with high affinity, followed by receptor integration and degradation, so that active cells substantially reduce the extracellular IL-2 concentration. In this way, Treg cells may outcompete Th cells for IL-2 and thus prevent sufficient stimulation of Th cells and initiation of a strong immune response (Busse et al., 2010; Feinerman et al., 2010; Fuhrmann et al., 2016; Oyler-yaniv et al., 2017; Thurley et al., 2015). Here, we show how key results of those studies can be obtained through response-time modeling.

Our first step is to characterize the input-to-output relationships of cell types. The response-time distribution for IL-2R up-regulation, as functions of the extracellular IL-2 concentration, could be obtained experimentally or even guessed. For simplicity, here we obtained the distributions computationally from an existing mathematical model, which considers IL-2R expression, degradation and binding to IL-2 molecules as intracellular building blocks (Figure B1B, Figure S5A, STAR Methods) (Busse et al., 2010). The resulting response-time distributions indicate that the probability to respond early increases with increasing IL-2 concentration, and is much higher for Treg cells than for Th cells (Figure B1C, Figure S5B). The second step is to model cell-cell interactions (Figure B1A). Here, we considered a previously characterized reaction-diffusion process of extracellular IL-2 secreted by Th cells and “consumed” by active cells via binding to IL-2R (Oyler-yaniv et al., 2017; Thurley et al., 2015), and we derived a simplified mathematical description (STAR Methods).

In this cell-cell-communication network, Treg cells effectively inhibit cell activation at weak-to-moderate antigen-induced IL-2 secretion rates, but Treg cells cannot prevent Th cell activation at strong stimulation. This can be directly observed from the response-time model (Figure B1D), recapitulating a “tug-of-war” scenario previously modeled (Feinerman et al., 2010) (note that despite qualitative agreement, that study differs, e.g., in the assumed IL-2 diffusion constant and therefore in the level of cell density required for activation). Finally, our response-time model recovers the steep all-or-none decision curve at high fractions of Treg cells, which was reported earlier based on extensive 3D multi-scale simulations (Figure B1E) (Thurley et al., 2015).

In summary, this response-time model unified and showed good qualitative agreement with previous, more detailed models. The approach did not rely on details of how the response-time distributions were generated and allowed us to abstract behaviors arising from intracellular dynamics and focus on cell population responses.
