# Supplementary material for: Modeling Cell-to-Cell Communication Networks Using Response-Time Distributions
Source: Cell Syst. Author manuscript; Available in PMC 2018 Apr 24. (PMC5913757; doi:10.1016/j.cels.2018.01.016)
Supplement: 1 [file NIHMS957759-supplement-1.pdf]

Cell Systems, Volume *6*

Supplemental Information

Modeling Cell-to-Cell Communication

Networks Using Response-Time Distributions

Kevin Thurley, Lani F. Wu, and Steven J. Altschuler

Supplemental Figures and Tables

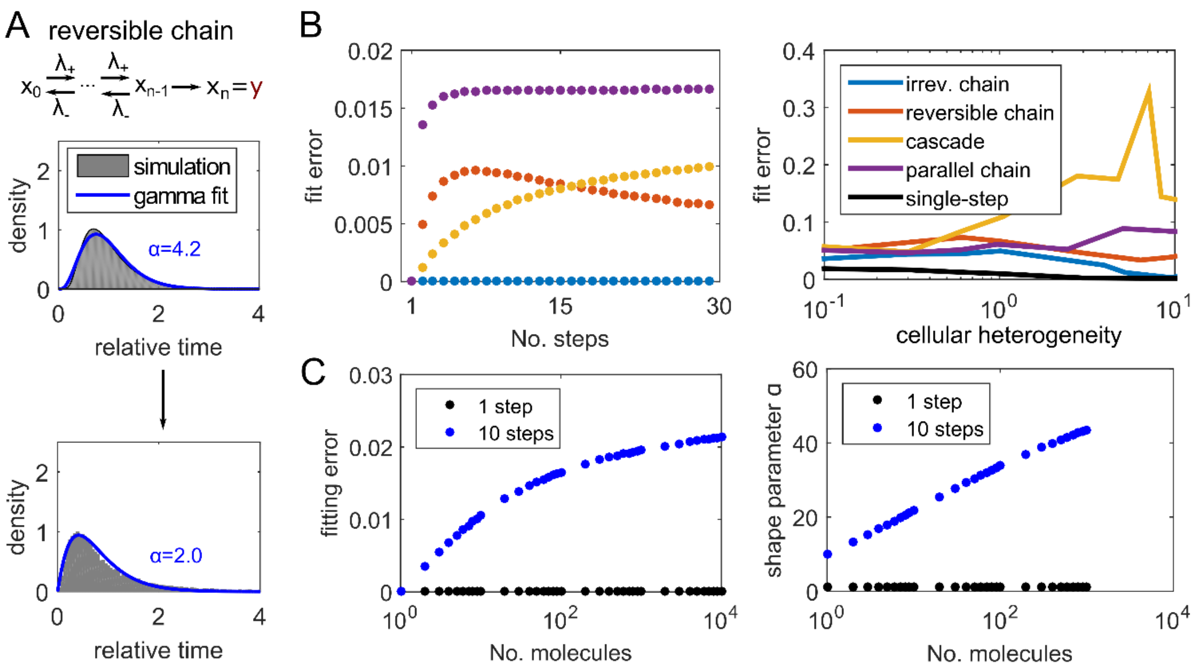

Figure S1: Supplementary analysis of simple multi-step models. Related to Figure 2.
(A) Analysis of the reversible chain model, analogous to Figure 2B-D.

(B) Fitting error (Root-mean squared sum of residuals) of best-fit gamma distributions to the indicated models (see Figure 2A-E), analogous to Figure 2G.

(C) Fitting error and shape parameter α for the parallel chain model (Figure 2D) with varying number of molecules (parameter m).

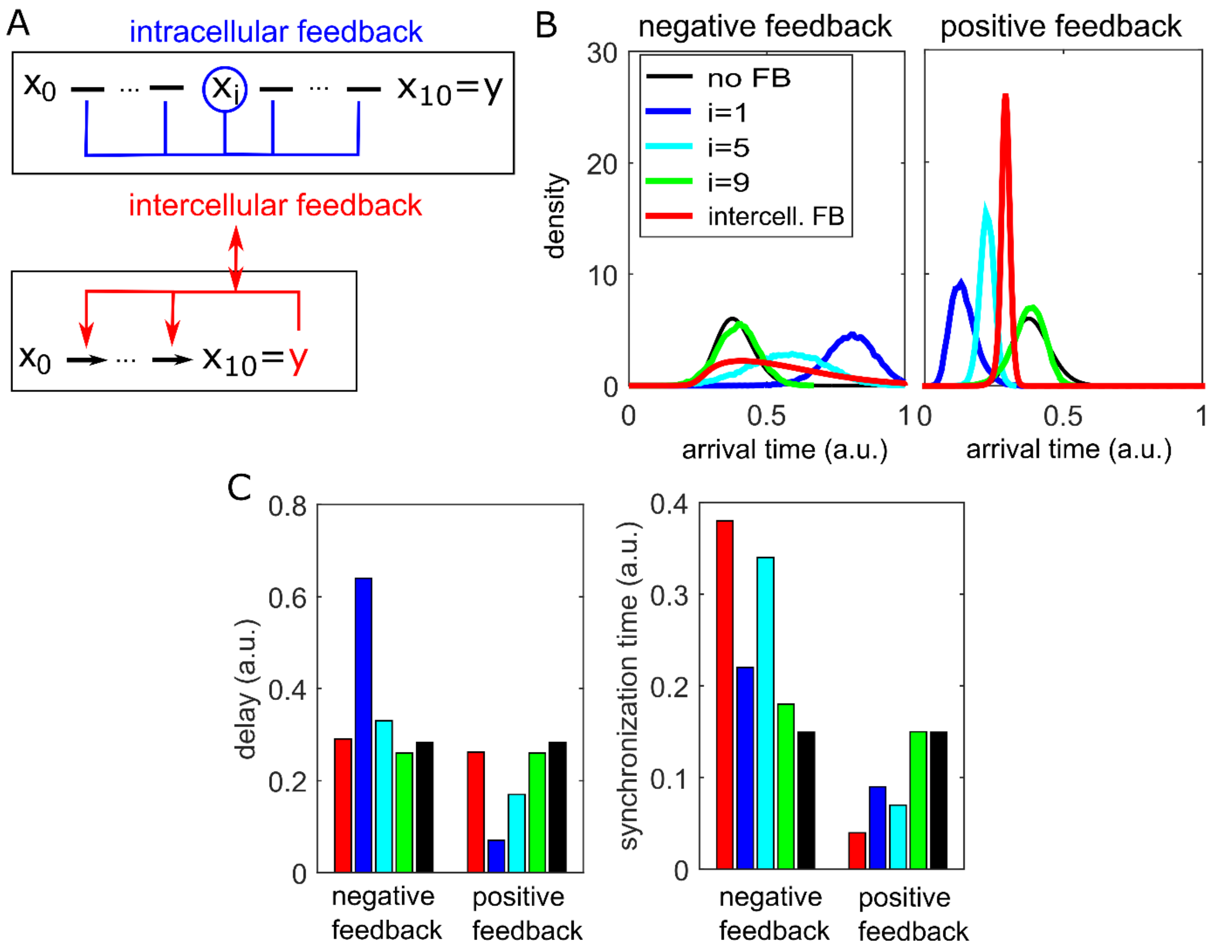

Figure S2: Comparison of intracellular and intercellular feedback. Related to Figure 3.
(A) Model scheme: The parallel chain model (Figure 2D; $n = 10$ steps, $m = 100$ molecules) is implemented either with inter-cellular feedback between cells (as in Figure 3B-D, left panels), or with intra-cellular feedback. The inter-cellular feedback is implemented by allowing molecules in the final state (here x_{10}) to alter all rate parameters in all cells. For intra-cellular feedback, the reaction rate depends on the number of molecules in an intermediate state x_i and there is no cell-cell communication.

(B-C) Arrival time distributions and values of synchronization time and delay for the indicated models: no feedback (FB); intracellular feedback (negative or positive) from state x_i with $i = 1,5,9$; intercellular feedback (negative or positive).

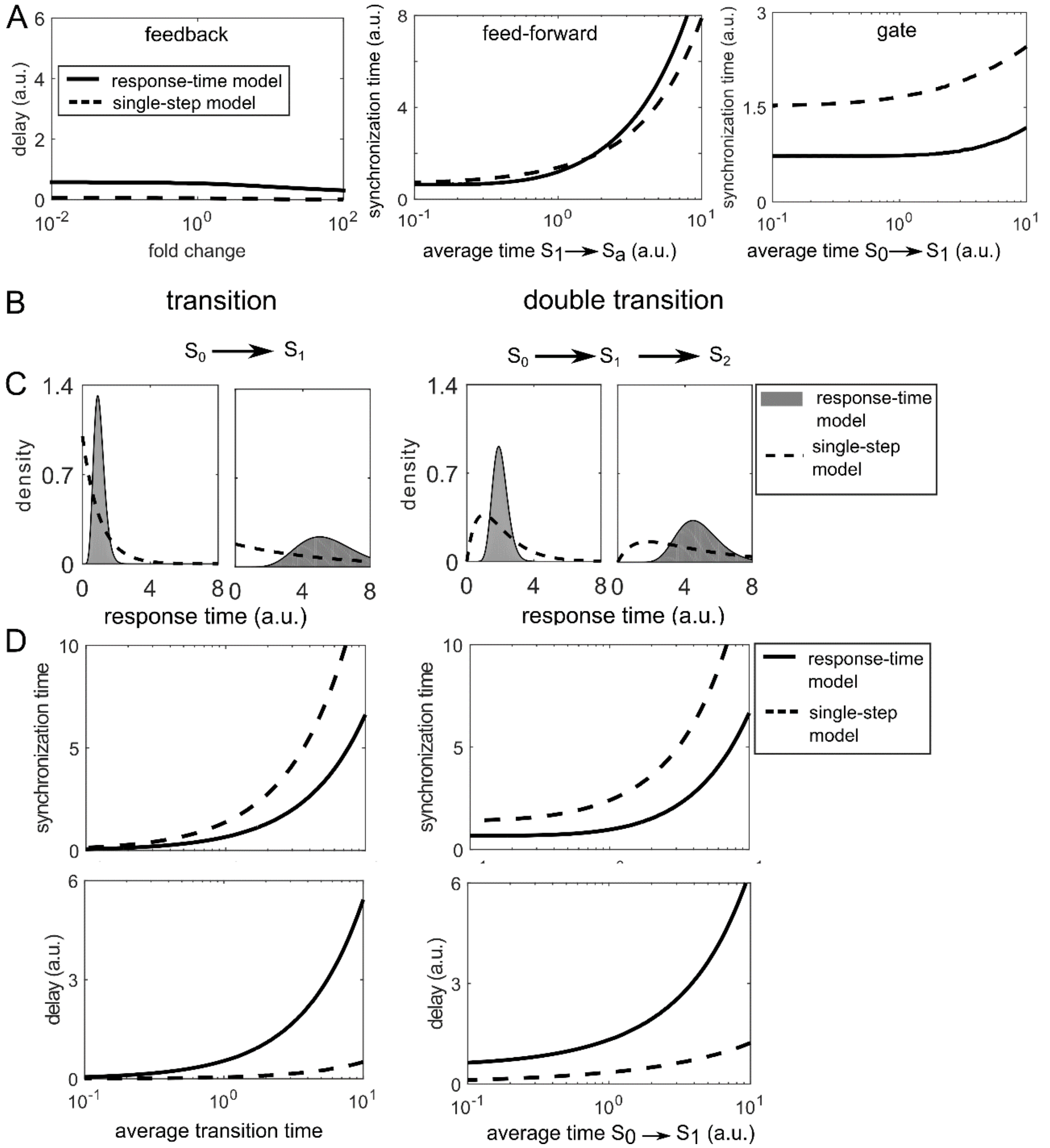

Figure S3: Supplementary analysis of intercellular interaction motifs. Related to Figure 3.
(A) Additional analysis of network motifs (see Figure 3B-D).

(B-D) Additional network motifs (see Figure 3B-D).

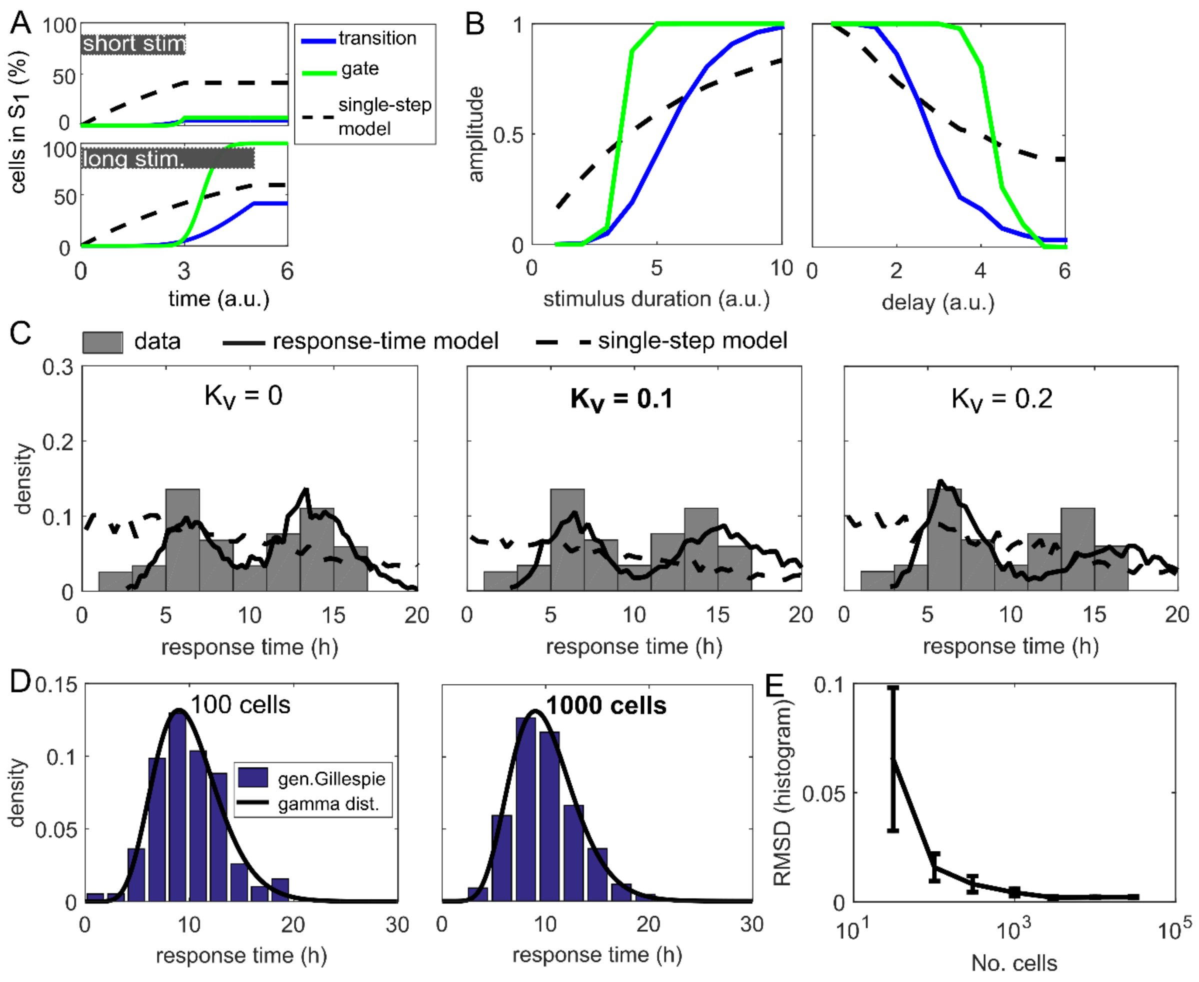

Figure S4: Supplementary analysis of response-time modeling applications. Related to Figure 5.

(A) Simulations are carried out with the best-fit gamma distributions (“response-time model) or exponential distributions (“single-step model”) to the arrival-time distributions arising in the gate and transition motifs (see Figure 4), all with the same delay value of $t_{\mathrm{delay}} = 3$. Black bars indicate stimulus duration.

(B) Maximal fraction of activated cells after stimulation (“Amplitude”) at varying stimulus duration (at $t_{\mathrm{delay}} = 3$) and delay (at duration $t_{\mathrm{d}} = 5$)(see Star Methods).

(C) Simulations of IFN-γ secretion onset (Figure 5E-G) with varying feedback strength K_v. Bold: Feedback strength used Figure 5G.

(D-E) Validation of the generalized Gillespie algorithm used in Figure 5E-G. (D) Simulations of the 10-step process with indicated number of cells, and comparison to the exact solution (gamma distribution, see Equation 1). (E) Root-mean-square deviation (mean and standard deviation from 4 simulations) in histograms such as shown in (D).

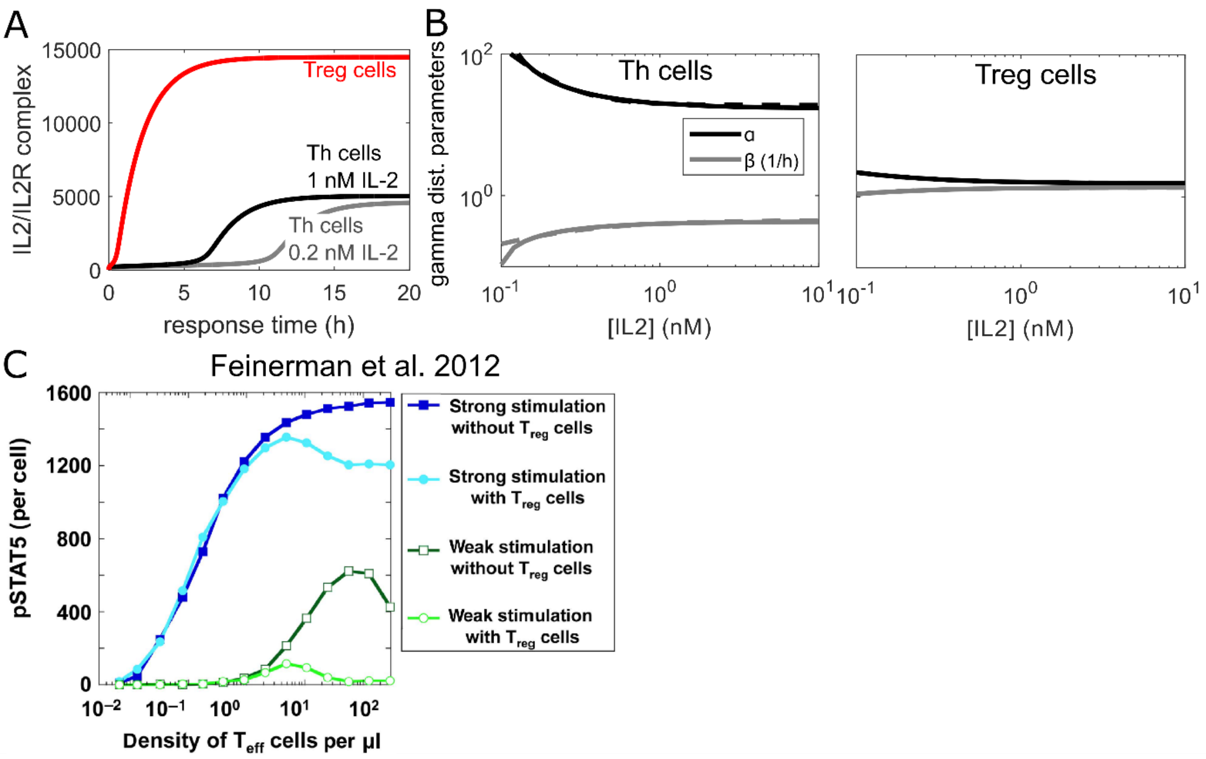

Figure S5: Supplementary data for IL-2 competition simulations. Related to Box 2.
(A) Simulation of the model shown in Box Figure, panel B, with indicated extracellular IL-2 concentration.

(B) Best-fit gamma distribution parameters α and β to curves as shown in Box Figure, panel C, which were obtained from simulations as shown in (A), for a range of IL-2 concentrations. Dashed lines are interpolating curves used for the implementation of the response-time model (see Star Methods).

(C) IL-2 competition (or "tug-of-war") simulation from (Feinerman et al., 2010), reprinted for comparison with simulations shown in Box Figure, panel D (right).

Table S1: Parameter values used in Figure 5.

Description	Value	Reference
Half-saturation constant of IL-2 interaction	0.05	
Shape parameter of CD25 up-regulation	34.9 (Figure 5E, bottom)	(Dorner et al., 2009)
Rate parameter of CD25 up-regulation	0.21 (Figure 5E, bottom)	(Dorner et al., 2009)
Shape parameter IL-2 secretion onset	4.33 (Figure 1A)	(Han et al., 2012)
Rate parameter IL-2 secretion onset	1.36 (Figure 1A)	(Han et al., 2012)
Shape parameter initial IFN-γ secretion onset	14.7 (Figure 1A)	(Han et al., 2012)
Rate parameter initial IFN-γ secretion onset	0.45 (Figure 1A)	(Han et al., 2012)
Fraction of IL-2+ cells	0.1	(Han et al., 2012)
Fraction of early IFN-γ+ cells	0.35	(Han et al., 2012)
Fraction of late IFN-γ+ cells	0.55	(Han et al., 2012)
Average duration of IL-2 secretion	4 hr	(Han et al., 2012)
Average duration of IFN-γ secretion	4 hr	(Han et al., 2012)

When figure panels are referred to, the parameter values are computed in those figures based on data in the cited reference.
